# Exosomes of Mesenchymal Stem Cells as a Proper Vehicle for Transfecting miR-145 into the Breast Cancer Cell Line and Its Effect on Metastasis

**DOI:** 10.1155/2021/5516078

**Published:** 2021-06-29

**Authors:** Mohsen Sheykhhasan, Naser Kalhor, Azar Sheikholeslami, Masoumeh Dolati, Elaheh Amini, Hoda Fazaeli

**Affiliations:** ^1^Department of Mesenchymal Stem Cells, Academic Center for Education, Culture and Research (ACECR), Qom Branch, Qom, Iran; ^2^Cellular and Molecular Research Center, Qom University of Medical Sciences, Qom, Iran; ^3^Department of Cellular & Molecular Biology, Faculty of Biological Sciences, Kharazmi University, Tehran, Iran

## Abstract

**Background:**

Despite recent advances in scientific knowledge and clinical practice, management, and treatment of breast cancer, as one of the leading causes of female mortality, breast cancer remains a major burden. Recently, methods employing stem cells and their derivatives, i.e., exosomes, in gene-based therapies hold great promise. Since these natural nanovesicles are able to transmit crucial cellular information which can be engineered to have robust delivery and targeting capacity, they are considered one of the modes of intercellular communication. miR-145, one of the downregulated microRNAs (miRNAs) in various cancers, can regulate tumor cell invasion, metastasis, apoptosis, and proliferation and stem cell differentiation.

**Objectives:**

The aim of this study was to investigate the role of exosomes secreted from adipose tissue-derived mesenchymal stem cells (MSCs) for miR-145 transfection into breast cancer cells in order to weaken their expansion and metastasis.

**Methods:**

Here, we exploited the exosomes from adipose tissue-derived mesenchymal stem cells (MSC-Exo) to deliver miR-145 in the T-47D breast cancer cell line. Lentiviral vectors of miR-145-pLenti-III-enhanced green fluorescent protein (eGFP) and empty pLenti-III-eGFP as the backbone were used to transfect MSCs and T-47D cells. In order to find the efficiency of exosomes as a delivery vehicle, the expression level of some miR-145 target genes, including Rho-Associated Coiled-Coil Containing Protein Kinase 1 (ROCK1), Erb-B2 Receptor Tyrosine Kinase 2 (ERBB2), Matrix Metalloproteinase 9 (MMP9), and Tumor Protein p53 (TP53), was compared in all treatment groups (T-47D cells treated by miR-145-transfected MSCs and their derivatives or their backbone) and control group (untransfected T-47D cells) using real-time PCR.

**Results:**

The obtained data represented the inhibitory effect of miR-145 on apoptosis induction and metastasis in both direct miR-treated groups. However, exosome-mediated delivery caused an improved anticancer property of miR-145.

**Conclusion:**

Restoration of miR-145 using MSC-Exo can be considered a potential novel therapeutic strategy in breast cancer in the future.

## 1. Introduction

Breast cancer is the second leading cause of female mortality that is considered a serious menace to women's health [1]. Regardless of tumor type, it is elucidated that typical cancer therapeutic modalities such as surgery, chemotherapy, and radiotherapy have their critical side effects and inefficiency in preventing disease recurrence [2]. Today, research studies on methods employing stem cells and their secretome including extracellular vesicles (EVs) have attracted great attention.

In this context, mesenchymal stem cells (MSCs) with the inherent capacity to migrate to tumor sites have attracted much attention as efficient tools to selectively deliver anticancer cargos to tumor sites. These multipotent stem cells can be isolated from several tissues such as the bone marrow, adipose tissues, cord blood, liver, and peripheral blood. In addition to having self-renewal potential and multipotent capacity, these capable secretory cells exert their paracrine effects via releasing EVs such as exosomes [3].

Exosomes are small vesicles (30-150 nm) with a bilayer lipid membrane and an endocytic origin that have received increasing attention in recent years for their association with biological and pathophysiological processes [4, 5]. Several investigations exhibited that due to their small size, exosomes may be utilized as delivery vehicles for tumor therapy without toxicity or immunogenicity risk associated with artificial carriers such as liposomes or nanoparticles. Exosomal microRNAs (miRNAs) secreted by tumor cells have been involved in the regulation of cancer formation and progression [6].

Over the past few decades, the ability of miRNA molecules in developing biopharmaceuticals against various cancers has been extensively studied [7]. miRNAs are short noncoding RNA molecules that regulate cellular pathways, including differentiation, proliferation, angiogenesis, and metabolism. miR-145 is among miRNAs that are downregulated in various cancers including ovarian, cervical, colorectal, and breast carcinomas [8]. It has shown that miR-145 can act as a tumor suppressor via suppression of breast cancer cell proliferation and invasion, and its low expression in breast tumors may be used as an efficient prognostic and diagnostic marker for patients [9]. Dong et al. in an in vitro study have shown that miR-145-5p sponging may induce breast cancer cell progression and metastasis via upregulating transforming growth factor-beta receptor 2 (TGFBR2) expression [10]. It induces its effects through direct and indirect modulation of several important genes, such as Matrix Metalloproteinase 11 (MMP11) [11], insulin receptor substrate 1 (IRS1) [12], homeobox A9 (HOXA9) [13], SRY-Box Transcription Factor 9 (SOX9), adducin 3 [14], ADAM metallopeptidase domain 17 (ADAM17) [15], octamer-binding transcription factor 4 (OCT4) [16], Kruppel-like factor 4 (KLF4) [17], ribosomal protein S6 kinase *β* 1 (also called p70S6K1 or S6K1) [18], c-Myc [19], mucin 1 [20], and Rho-Associated Coiled-Coil Containing Protein Kinase 1 (ROCK1) [11]. Also, in a study by Pan et al., it was demonstrated that miR-145 could reduce angiogenesis of human umbilical vein endothelial cells (HUVECs) via IRS1/phosphoinositide 3-kinase (PI3K)/Akt kinase (Akt)/Mechanistic Target of Rapamycin (mTOR) and IRS1/proto-oncogene serine/threonine kinase (Raf)/extracellular signal-regulated kinase (ERK) pathways through directly repressing IRS1 [21].

Growing pieces of evidence suggest that there is an interaction between stem cells and human tumor cells in the exchange of biological information through exosomes. These EVs naturally contain genetic materials such as miRNAs and can transfer their content to recipient cells and exert their phenotypic effects by playing multiple roles in tumorigenesis, angiogenesis, and tumor metastasis [22].

EVs can resolve the most common safety considerations correlated with direct cell transplantation, such as the risk of embolism due to intravascular injection, pathological or tumor transformation due to genetic disorders or uncontrolled cell differentiation, or immune activation for allogeneic preparations [23]. Besides, unlike transplanted cells that cannot be recovered, treatment with EVs is not permanent and can be easily stopped if adverse effects occur. Moreover, produced EVs can be evaluated for their safety, dosage, and potential by common methods used for other pharmacological agents so that they can quickly enter the clinical pathway. Furthermore, purified EVs can be stored for long periods with no harm to their biological activity (-20 degrees for six months [24] or -80 degrees for more than two years [25]). As a result, the possibility of their storage and transmission presents EVs as off-the-shelf therapeutic agents.

The increasing evidence may verify the ability of exosomes as effective options for transferring miRNAs to targeted cells and confirm the suppressor potential of miR-145 against various cancer types, especially breast cancer. Hence, miR-145 delivery using mesenchymal stem cell- (MSC-) derived exosomes can be considered a theory that in practice may reduce the proliferation and metastasis abilities in breast cancer cells. According to the mentioned hypothesis, this study is aimed at investigating the role of exosomes secreted from adipose tissue-derived MSCs for miR-145 transfection into breast cancer cells in order to weaken their expansion and metastasis.

## 2. Material and Methods

### 2.1. Ethics Statement and Study Design

This interventional experimental study was approved by the ACECR Biomedical Research Ethics Committee (IR.ACECR.JDM.REC.1398.003). In this study, we evaluated the efficacy of exosomes as a delivery carrier of microRNAs in comparison to direct transfection or use of adipose-derived mesenchymal stem cells (AD-MSCs) or their conditioned medium. The study was designed in 10 groups of T-47D breast cancer cells which were defined as follows:

1. Control group (untransfected T-47D cells)
2. miR group (transfected T-47D cells with miR-145)
3. BB group (transfected T-47D cells with BB)
4. MSC group (coculture of T-47D cells with MSCs)
5. miR-MSC group (coculture of T-47D cells with transfected MSCs with miR-145)
6. BB-MSC group (coculture of T-47D cells with transfected MSCs with BB)
7. miR-CM-MSC group (treatment of T-47D cells with CM of transfected MSCs with miR-145)
8. BB-CM-MSC group (treatment of T-47D cells with CM of transfected MSCs with BB)
9. miR-MSC-Exo group (treatment of T-47D cells with exosomes of transfected MSCs with miR-145)
10. BB-MSC-Exo group (treatment of T-47D cells with exosomes of transfected MSCs with BB)

**Figure 1:**
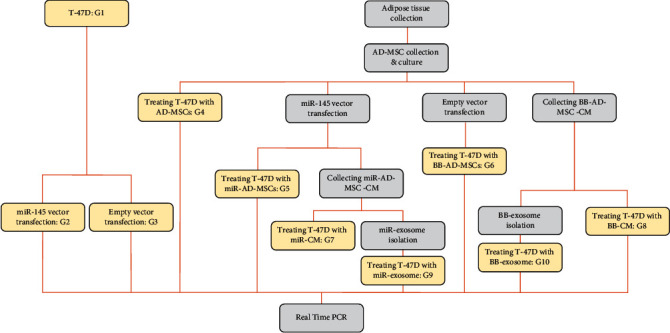
The flowchart of the study design.

The study procedure is shown in the following flowchart (Figure 1). Finally, to study the resulting changes in different groups and also to prove cell transfection, the expression of miR-145, ROCK1, Erb-B2 Receptor Tyrosine Kinase 2 (ERBB2), and Matrix Metalloproteinase 9 (MMP9) as involved genes in metastasis and Tumor Protein p53 (TP53) as an apoptotic gene was assessed using the real-time PCR method.

### 2.2. Isolation and Culture of AD-MSCs

Written informed consent was taken from volunteer patients undergoing liposuction surgery to collect adipose tissue samples which are considered surgical waste material. The AD-MSCs were characterized and isolated according to our previous study using the same isolation and culture protocol of AD-MSCs in which cells were confirmed through assessing morphology, surface marker flow cytometry, and triple differentiation ability into chondrocyte, osteocyte, and adipocyte lineages [26]. Briefly, the adipose tissue samples were mechanically segmented and then rinsed with phosphate-buffered saline (PBS) several times. Enzymatic digestion was performed using collagenase type I (1.5 mg/g) at 37°C for 45-60 min. Then, the cell suspension was centrifuged at 1800 revolutions per minute (rpm) for 10 min and the cell pellet was cultured in Dulbecco's Modified Eagle's Medium (DMEM) supplemented with 1% penicillin-streptomycin and 10% Fetal Bovine Serum (FBS) and incubated overnight in plastic culture dishes, followed by a washing step to remove nonadherent cells. The medium was changed at least twice per week until the cells reach up to 80% confluence. For treatments, the cells were used in passage 3.

### 2.3. Culture of the T-47D Cell Line

The human breast cancer cell line T-47D was obtained from Pasteur Institute (Tehran, Iran), and cells were cultured in DMEM/Ham's F12 supplemented with 10% FBS and 1% penicillin/streptomycin. Cells were grown in a humidified atmosphere of 95% air and 5% CO_2_.

### 2.4. Lentiviral Transfection of T-47D Cells and AD-MSCs

**Figure 2:**
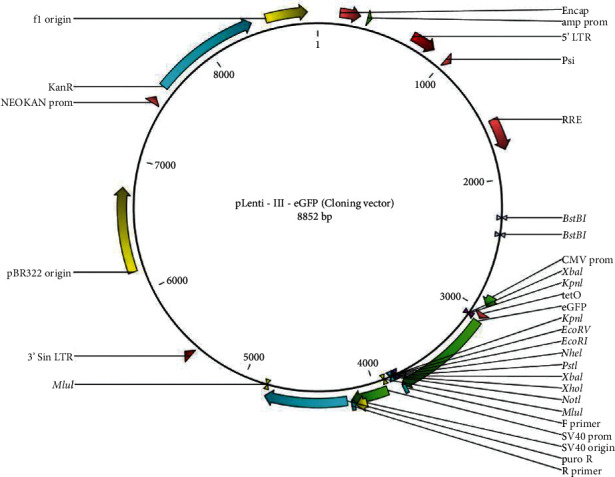
Map of miR-145-pLenti-III-eGFP.

Lentiviral vectors of miR-145-pLenti-III-eGFP and empty pLenti-III-eGFP as the backbone were purchased from Stem Cells Technology Research Center, Iran (Figure 2), and transfection was done through a modified calcium phosphate method. Briefly, 24 h before transfection, cells were harvested at a density of 3 × 10^5^ cells/cm^2^ in 6-well plates. The cells were then incubated at 37°C in a 90% humidified incubator and 5.5% CO_2_. The culture medium was changed 3 h before transfection. To prepare the calcium phosphate-DNA coprecipitate, 100 *μ*l of 2.5 M CaCl_2_ with 30 *μ*g of plasmid DNA was added in a sterile microtube, and the final volume was increased to 1 ml with 0.1x TE (pH 7.5). Then, the calcium phosphate-DNA solution was slowly (within 1 min) added into a tube containing 1 ml 2x HEPES-buffered saline (HBS) and the solution was incubated at 37°C for 25 min. After that, the suspension was immediately transferred into the medium above the cell monolayer (0.1 ml of the suspension was used for every 1 ml of the medium). After incubating cells at 37°C for 5 h, the medium was aspirated and the cells were washed by PBS solution. In this stage, 4 treatments were administered to find the optimized calcium phosphate method: in group 1, 1 ml of 15% glycerol was added, and after incubation of cells at 37°C for 45 s, the glycerol was removed by aspiration, and cells were washed by PBS, while in group 2, the incubation time was 30 s. Removing glycerol and washing with PBS were eliminated in group 3, but in group 4, glycerol was not added at all. The rest of the procedure was the same in all 4 groups: the culture medium without the puromycin antibiotic was added. Two days after incubation, the medium was replaced by a puromycin-containing medium. The medium was changed every 1-4 days for 2-3 weeks, and then transfection was assessed using an inverted fluorescence microscope.

### 2.5. Collection of the AD-MSC Conditioned Medium (CM-MSC)

To collect the conditioned medium, AD-MSCs were seeded (at passage numbers 3-4) in T-75 flasks at a density of 1 × 10^6^ cells. Two days after reaching 70–80% confluence (8 × 10^5^ cell/cm^2^), the CM was collected, centrifuged, and filter sterilized through a 0.22 *μ*m filter. The collected CM was stored at −70°C.

### 2.6. Exosome Isolation of AD-MSCs (MSC-Exo)

To exosome isolation, the medium of AD-MSCs was replaced with the medium containing less FBS every three days. After a gradual decrease in the serum level to zero, exosomes were isolated from FBS-free CM based on the protocol of the Exocib kit (Cib Biotech Co.). Briefly, the filtered CM was mixed with the exosome precipitation solution and incubated overnight at 4°C. The samples were centrifuged for 40 min at 3000 rpm. Then, the supernatants were discarded, and the pellet was resuspended with PBS. Finally, the purified exosome samples were stored at −70°C. The protein content of the AD-MSC exosomes was determined using a Bicinchoninic Acid (BCA) protein assay kit (Sigma-Aldrich, Missouri, USA).

### 2.7. Characterization of MSC-Exo

#### 2.7.1. Transmission Electron Microscopy of Exosomes

The isolated exosomes were fixed with 4% paraformaldehyde, applied onto formvar-coated carbon grids, and stained with 1% phosphotungstic acid at RT for 2 min. The morphological features of isolated exosomes were finally observed using an FEI Tecnai G2 Spirit transmission electron microscope (TEM) (Thermo Fisher Scientific, Inc.) [27].

#### 2.7.2. Flow Cytometric Analysis of Exosomes

The isolated exosomes were characterized using 0.1 *μ*m polystyrene beads to adjust the instrument voltages, and then they were labeled with the anti-human antibodies, including anti-CD81 and anti-CD63 antibodies, for flow cytometric analysis (both antibodies were purchased from eBioscience). The analysis was carried out using a FACSCalibur flow cytometer.

#### 2.7.3. Dynamic Light Scattering of Exosomes

Dynamic light scattering (DLS) is a technique in which the size distribution of the particles that have a diameter ranging from 1 nm to 6 *μ*m can be measured. The particles are illuminated with a laser beam. So, all vesicles present in the beam will disseminate light. The intensity fluctuations of the disseminated light will be measured, and a mathematical model derived from Brownian motion and light scattering theory will be applied. So, the size distribution of these vesicles is assessed [28]. For this aim, samples were diluted to 1 *μ*g/ml in PBS and 0.05% Tween-20, and the size of them was evaluated by DLS Zetasizer Nano ZS (Malvern Instruments, UK).

### 2.8. Coculture of T-47D Cells with MSCs

T-47D cells were seeded in 6-well plates at a density of 8 × 10^4^ cells per well. Then, 3 × 10^4^ AD-MSCs were cultured on T-47D cells in each well and incubated at 37°C for 72 h in a 90% humidified incubator and 5% CO_2_. The cells were culture in DMEM supplemented with 1% penicillin-streptomycin and 10% FBS.

### 2.9. Treating T-47D Cells with CM-MSCs

After centrifugation of the collected MSC CM for 5 min at 2000 rpm, it was added to T-47D cells seeded in 6-well plates at a density of 8 × 10^4^ cells per well. Cells were incubated for 72 h in the same condition mentioned as coculture of MSCs and T-47D cells.

### 2.10. Treating T-47D Cells with MSC-Exo

T-47D cells were seeded in 12-well plates at a density of 4 × 10^4^ cells per well. Then, the isolated exosomes were added to serum-free DMEM containing 1% penicillin-streptomycin per well. The cells were incubated for 72 h at 37°C in a 90% humidified incubator and 5% CO_2_.

### 2.11. Real-Time PCR

Total RNA was isolated from cells using the GeneAll Kit (GeneAll Biotechnology, Seoul, Korea) according to the manufacturer's instructions. RNA purity and quantity were assessed using a NanoDrop 2000 spectrophotometer (Thermo Fisher Scientific, Wilmington, USA) at 260/280 nm. The reverse transcription was used to synthesize the first-strand cDNA using a transcription kit (Yekta Tajhiz, Iran). Quantitative real-time PCR assays were performed in triplicate to evaluate the expression of miR-145, ROCK1, Matrix Metalloproteinase 9, p53, and epidermal growth factor receptor (EGFR). Since the glyceraldehyde-3-phosphate dehydrogenase (GAPDH) gene is often stably and constitutively expressed at high levels in most tissues and cells, it was considered a housekeeping gene in order to normalize gene expression levels. The 2^-*ΔΔ*CT^ method was used to calculate the fold change of mRNA expression. Real-time PCR was carried out using RealQ Plus Master Mix Green (Ampliqon III) following the manufacturer's instructions. Briefly, the mixture composed of 10 *μ*l SYBR green mix, 1 *μ*l cDNA (250 ng), 1 *μ*l PCR forward primers and 1 *μ*l PCR reverse primers in 5 pmol/*μ*l, and Millipore water was added to achieve a final volume of 20 *μ*l. The sequences of primers are presented in Table 1. The Threshold Cycle (CT) was determined manually for each run. The relative mRNA level was expressed as the relative fold change and calculated using the formula 2^–ΔΔCT^ = 2^–(ΔCT(sample) − ΔCT(calibrator))^, where each ΔCT = ΔCT Target–ΔCT GAPDH. One sample without any treatment from the control group was designated as a calibrator. The quantification of mRNA was performed as a value relative to an internal reference for GAPDH.

## 3. Results

### 3.1. AD-MSC Characterization

**Figure 3:**
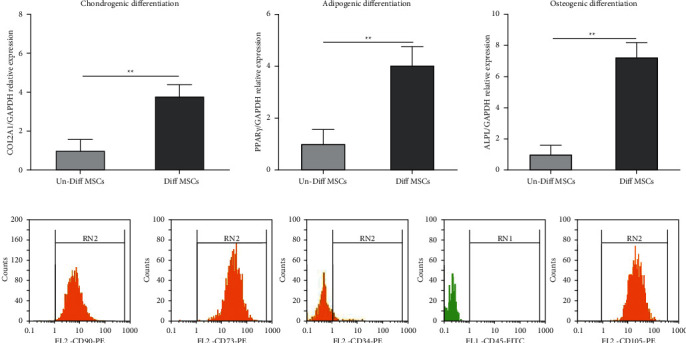
Characterization and differentiation of AD-MSCs. (a) mRNA expression level of chondrogenic, adipogenic, and osteogenic markers assessed after two weeks by real-time PCR analysis in differentiated and undifferentiated AD-MSCs. (b) AD-MSC surface marker expression, including CD90, CD73, CD105, CD34, and CD45.

In our study, based on microscopic observations, AD-MSCs showed fibroblast-like spindle-shaped morphology, and according to flow cytometric assay, they were positive for CD73, CD90, and CD105 and negative for CD34 and CD45 in the 3rd passage. Also, the potential of chondrogenic, osteogenic, and adipogenic differentiation was assessed by the real-time PCR procedure. It was found that in comparison to undifferentiated AD-MSCs, the expression level of PPAR*γ*, collagen type II, and alkaline phosphatase genes was significantly higher in differentiated cells into adipocyte, chondrocyte, and osteocyte lineages, respectively (*p* = 0.008, *p* = 0.003, and *p* = 0.001, respectively) (Figure 3).

### 3.2. Characterization of MSC-Exo

**Figure 4:**
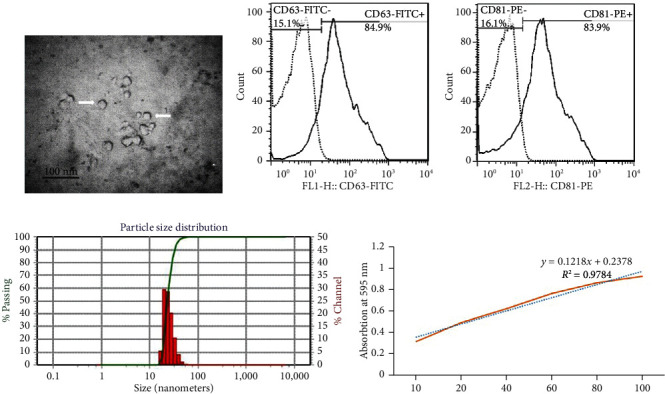
(a) Electron micrograph image of AD-MSC exosomes (white arrows indicate exosomes). (b) FACS analysis of CD81 and CD63 expressed on the surface of AD-MSC exosomes. (c) Dynamic light scattering of MSC exosomes for measuring the mean size of these nanoparticles. (d) BCA protein assay of AD-MSC exosomes, where absorbance was measured at 595 nm.

Characterization of the shape and size of the isolated exosomes was carried out by TEM, DLS, and flow cytometric assay for CD81 and CD63. Among the EVs, the size of the exosomes is between 50 and 150 nm [29], and in our study, as was expected, the mean size of isolated exosomes was within this range (Figure 4).

### 3.3. Enrichment of miR-145 in T-47D Cells, AD-MSCs, and Their Derivatives

**Figure 5:**
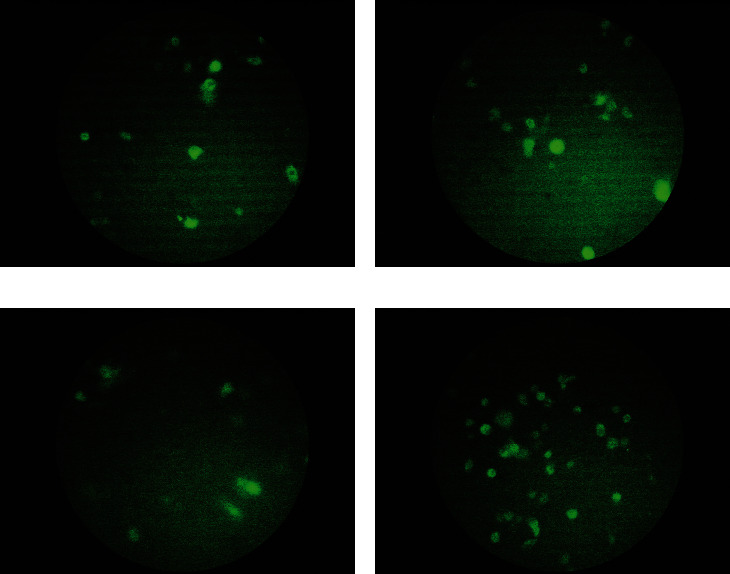
Fluorescence microscopy images of different modifications of the calcium phosphate lentiviral transfection method. (a) Group 1: incubation with glycerol for 30 s followed by washing with PBS. (b) Group 2: incubation with glycerol for 45 s followed by washing with PBS. (c) Group 3: eliminating the glycerol adding and PBS washing steps. (d) Eliminating the glycerol adding step.

Successful lentiviral transfection of cells was confirmed through visualization of green fluorescent protein (GFP) expression using fluorescence microscopy. As it is shown in Figure 5, transfection in group 3, in which no glycerol was used, showed more efficiency, so that this modification was chosen to be applied in our study.

**Figure 6:**
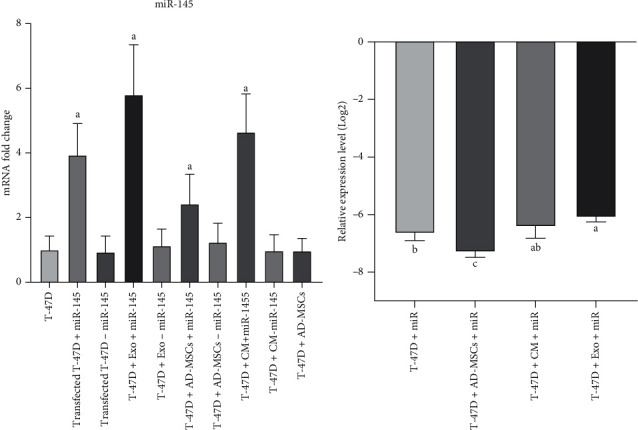
(a) The evaluation of the miR-145 level expression in transfected T-47D cells and groups treated with miR-MSCs or their derivatives in comparison to the control group. (b) Assessing the efficacy of miR-145 transfection among groups. miR: miR-145; CM: conditioned medium; Exo: exosome. *p* ≤ 0.005.

The expression level of miR-145 was assessed through qRT-PCR in all groups that received direct or indirect (by transfected AD-MSCs or their derivatives) miR-145 via lentiviral transfection. A significant increase in miR-145 was verified in the miR and miR-MSC groups in comparison to the control group (3.93- and 2.42-fold, *p* = 0.007 and *p* = 0.01, respectively). Also, a 5.79-fold increase in miR-145 was detected in the miR-MSC-Exo group (*p* < 0.0001), while analysis of the miR-CM-MSC group revealed a significant (4.64-fold) increase in expression of miR-145 compared to the control group (*p* < 0.0001) (Figure 6(a)).

Furthermore, in the case of transfection efficacy, statistical analysis showed that while exosomes were significantly more efficient in transfecting T-47D cells than direct transfection (*p* = 0.033), no significant difference was observed in comparison to the miR-CM-MSC group (*p* = 0.179). Similarly, a significant priority in transfection was observed for the miR and miR-CM-MSC groups in comparison to the MSC group (*p* = 0.012 and *p* = 0.003, respectively), while the efficacy of direct transfection and use of miR-CM-MSCs were not significantly different (*p* = 0.302) (Figure 6(b)).

### 3.4. qRT-PCR Analysis

Based on the previous studies, since low expression of miR-145 is associated with cell proliferation and migration in a wide variety of tumors, we evaluated the expression level of some related genes including ROCK1, ERBB2, MMP9, and p53 in all 9 experimental groups in comparison to the control group. Furthermore, to assess the efficacy of affected groups, they were statistically compared to each other, as well. The sequences of all the designed primers are shown in Table 1. The efficiency of the primers and the PCR tests were assessed using LinRegPCR software.

### 3.5. Assessing the MMP9 Expression Level

**Figure 7:**
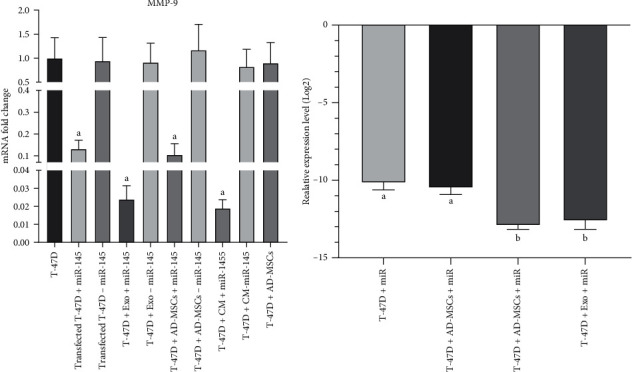
Statistical analysis of the MMP9 gene expression level. (a) Comparing the expression of the MMP9 gene between all treated groups and the control group. (b) Assessing the efficacy of significantly affected groups. miR: miR-145; BB: backbone; CM: conditioned medium; Exo: exosome. *p* ≤ 0.005.

A significant decrease in MMP9 expression was observed not only in the miR group (7.69-fold) but also in all groups treated with either miR-MSCs (9.09-fold) or their derivatives (conditioned medium and exosomes, 50- and 41.67-fold, respectively) (*p* < 0.0001), while in their corresponding backbone, there was no significant change in comparison to the control group (*p* > 0.05) (Figure 7(a)). When the affected groups were statistically compared to each other, we found that in both the miR-CM-MSC and miR-MSC-Exo groups, the expression level of MMP9 was more efficiently decreased in comparison to the miR and miR-MSC groups (*p* < 0.0001 and *p* = 0.001; *p* < 0.0001 and *p* = 0.001, respectively), while there was no significant difference either between the direct miR and miR-MSC groups (*p* = 0.792) or between the miR-CM-MSC and miR-MSC-Exo groups (*p* = 0.821).

### 3.6. Assessing the TP53 Expression Level

**Figure 8:**
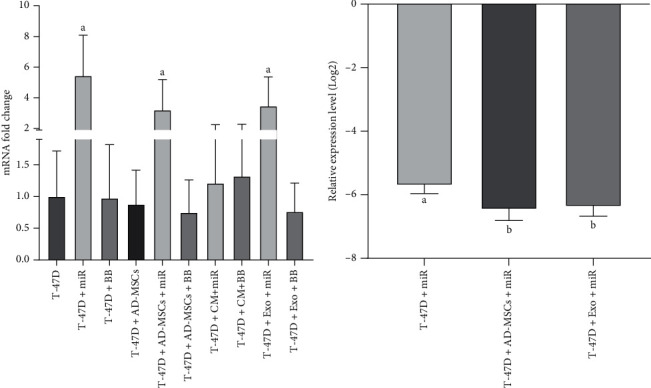
Statistical analysis of the TP53 gene expression level. (a) Comparing the expression of the TP53 gene between all treated groups and the control group. (b) Assessing the efficacy of significantly affected groups. miR: miR-145; BB: backbone; CM: conditioned medium; Exo: exosome. *p* ≤ 0.005.

As it is shown in Figure 8(a), the expression level of TP53 in the miR group was 5.45-fold higher than that in the control group (*p* < 0.0001). Similarly, an approximately 3-fold increase in gene expression was observed in the miR-MSC and miR-MSC-Exo groups in comparison to the control group (*p* = 0.007 and *p* = 0.009, respectively), while the increased expression in the miR-CM-MSC group did not reach a significant level (*p* = 0.915). Also, in all the backbone groups (BB, BB-MSC, BB-CM-MSC, and BB-MSC-Exo), TP53 expression was not significantly changed (*p* > 0.05). On the other hand, among the significantly changed groups, the expression level of TP53 was significantly higher in the miR-MSC and miR-MSC-Exo groups in comparison to the miR group (*p* = 0.029 and *p* = 0.047, respectively) (Figure 8(b)).

### 3.7. Assessing the ERBB2 Expression Level

**Figure 9:**
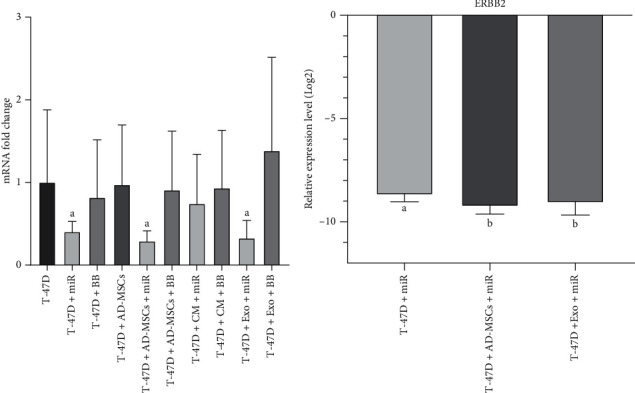
Statistical analysis of the ERBB2 gene expression level. (a) Comparing the expression of the ERBB2 gene between all treated groups and the control group. (b) Assessing the efficacy of significantly affected groups. miR: miR-145; BB: backbone; CM: conditioned medium; Exo: exosome. *p* ≤ 0.005.

The obtained results showed a significant decrease of the ERBB2 expression level in the miR (2.5-fold), miR-MSC (3.45-fold), and miR-MSC-Exo (3.03-fold) groups in comparison to the control group (*p* = 0.005, *p* < 0.0001, and *p* = 0.001, respectively). But in the case of miR-CM-MSCs and all backbone groups, no significant change in the ERBB2 expression level was observed (*p* > 0.05) (Figure 9(a)). More statistical analyses revealed that in both miR-MSCs and miR-MSC-Exo, the expression level of ERBB2 was more efficiently decreased in comparison to the miR group (*p* = 0.023 and *p* = 0.045, respectively) (Figure 9(b)).

### 3.8. Assessing the ROCK1 Expression Level

**Figure 10:**
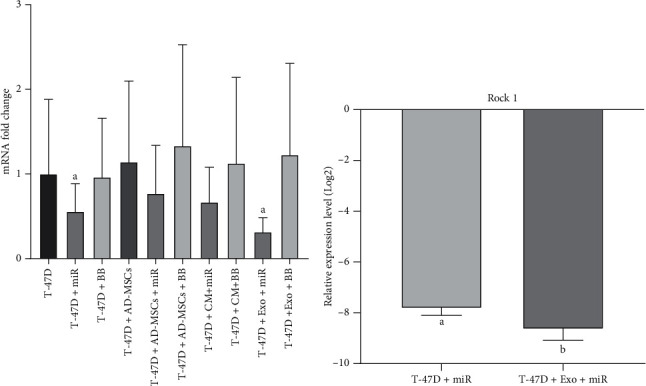
Statistical analysis of the ROCK1 gene expression level. (a) Comparing the expression of the ROCK1 gene between all treated groups and the control group. (b) Assessing the efficacy of significantly affected groups. miR: miR-145; BB: backbone; CM: conditioned medium; Exo: exosome. *p* ≤ 0.005.

Based on the obtained data, a significant increase in the ROCK1 expression level was observed only in the miR (*p* = 0.04) and miR-MSC-Exo (*p* < 0.0001) groups in comparison to the control group (1.79- and 3.13-fold, respectively), while not only all backbone groups but also the miR-MSC and miR-CM-MSC groups did not show a significant change in the ROCK1 expression level when compared to the control group (*p* > 0.05) (Figure 10(a)). Also, as it is shown in Figure 10(b), the efficacy of the miR-MSC-Exo group in decreasing the ROCK1 expression level was significantly higher than that of the miR group (*p* = 0.047) (Figure 10(b)).

## 4. Discussion

Since the discovery of the miRNAs in the 1990s, their role as master modulators of the human genome through posttranscriptional mechanisms, including either binding to the 3′-untranslated region (3′-UTR) of gene and translation repression or mRNA degradation, has been well documented [30].

Several studies have been conducted to evaluate the basic biogenesis and function of various miRNAs in different cancer-associated biological processes such as proliferation, differentiation, apoptosis, metabolism, invasion, metastasis, and drug resistance [31]. Further, miRNA profiling studies have identified miRNAs that are dysregulated in multiple cancer types [32]. As the second most common cancer among women, breast tumors and their aberrantly expressed oncogenic or tumor-suppressive miRNAs have attracted great scientific interest.

Among identified miRNAs, miR-145—located in a cluster within the 5q32-33 chromosomal region—is significantly downregulated in the patient's breast tumor tissues compared to healthy breast tissues [33]. In a recent study on the epigenetic state of the miR-145 promoter, methylation of the miR-145 promoter has been reported as a probable mechanism for its reduced expression, while demethylation leads to inhibition of migration and invasion of breast cancer cells through targeting the angiopoietin 2 gene [34].

Since systemic cytotoxic agents as the basis of current treatments usually cause severe side effects, there is a great potential for applying tumor-suppressive miRNAs like miR-145 to develop targeted therapies for breast cancer metastasis treatments [35]. However, the development of the most efficient delivery method to target cancer cells and tumor tissues is a major challenge for successful, nontoxic, and safe clinical applications of promising miRNA-based therapeutics. Due to the low stability of naked miRNAs against nucleases, the use of a delivery system that protects them from early degradation seems to be necessary [36]. Moreover, biocompatibility and biodegradability of delivery materials, having low immunogenicity, effective distribution to the target cells, and facilitating targeted uptake are the other criteria that should be considered during the development of an ideal delivery system [37].

Since exosomes can keep their content and are fully functional during transferring to recipient cells, a large number of studies have recently focused on the potential of exosome-mediated delivery of miRNAs or anti-miRNAs as a cancer therapy. Recent research studies, especially in breast cancer, have highlighted a promising therapeutic potential for exosomal delivery, whereas it was shown that in vitro enrichment of exosomes by miR-134 or miR-503 can impair cellular invasion, migration, and proliferation [38, 39].

In this study, we used MSC-derived exosomes as the carrier of miR-145 to treat T-47D cells and the obtained results were compared with direct transfection or application of miR-transfected AD-MSCs or their conditioned medium. Our findings frequently confirmed the tumor-suppressive effects of miR-145 by modulating ROCK1, MMP9, ERBB2, and TP53 gene expression. Also, its ability to regulate cell growth and proliferation through targeting and silencing c-Myc—a well-known oncogene—has been reported both in vitro and in vivo [40]. On the other hand, due to the silencing of metastasis gene mucin 1 (MUC1), it is demonstrated that miR-145 can inhibit the invasion ability of metastatic breast cancer cell lines. In a more recent study, Ding et al. showed that transforming growth factor-beta (TGF-*β*) protein expression may be inhibited by miR-145 overexpression which in turn prevents tumor formation [9].

MMP9, a 92 kDa protein with protease activity, is one of the most active enzymes in the MMP family for the degradation of type IV collagen and plays an important role in tumor vascularization, invasion of tumor cells, and metastasis [41]. The role of MMP9 in activating and proliferating breast cancer cells through collagen digestion and association with tumor-suppressive genes has been indicated in studies. Extracellular matrix degradation by MMP proteins facilitates cancer cell migration [42, 43]. Furthermore, the extracellular cell-cell and cell-matrix junctions are disrupted by these proteins which leads to separating tumor cells from epithelial sheets and activating signaling pathways that cause widespread changes in the pattern of gene expression involved in migration and invasion [44, 45].

The expression of MMP2 and MMP9 was detected in the SGC-7901 cell line in a study by Wang et al., which focused on the role of miR-145 in gastric cancer. It was shown that these MMP proteins were decreased in miR-145-transfected SGC-7901 cells [46]. Moreover, recently, the downregulation of MMP9 in the A549 lung cancer cell line transfected with miR-145 has been reported, suggesting that miR-145 replacement in lung cancer can be used to suppress the tumor metastasis [47]. Therefore, the expression level of MMP9 was evaluated in different groups of the present study. In line with previous studies and as it is illustrated in Figure 7(a), MMP9 in all groups receiving miR-145 was significantly downregulated. Interestingly, both the miR-CM-MSC and miR-MSC-Exo groups caused more efficient delivery of miR-145, and subsequently, a significant decrease in the MMP9 expression level was seen when compared to the control and miR-MSC groups.

The ERBB2 gene (also known as human epidermal growth factor receptor 2 (HER2)) has a critical role in human malignancies, and in about 30 percent of breast cancers, it is exacerbated or overexpressed [48, 49]. This gene encodes a 185 kDa transmembrane glycoprotein (p185ERBB2) with intrinsic tyrosine kinase activities which belong to the EGFR family [50–54]. In fact, overexpression of ERBB2 was shown to facilitate invasion and metastasis of breast cancer and to associate with low overall survival [55, 56]. In a research study performed by Khan et al., it was confirmed that in pancreatic cancer cells and human tumor tissues, miR-145 expression inversely associates with mucin 13 (MUC13) expression. The researchers revealed that MUC13 is targeted by miR-145, and so its protein expression is downregulated. Thus, miR-145 overexpression can prevent cell proliferation and invasion, which leads to an increase in p53 and a decrease in HER2, p21 (RAC1) Activated Kinase 1 (PAK1), and Phospho-Akt (P-Akt) expression [57]. Moreover, miR-145 injections in xenograft mice suppressed tumor growth through inhibition of MUC13 and HER2 as its downstream target [57].

In the present study, the expression level of ERBB2 was significantly decreased in all direct or indirect miR-145-transfected groups except the miR-CM-MSC group. Among the affected groups, the miR-MSC-Exo and miR-MSC groups show more efficiency in decreasing ERBB2 expression levels.

Some research studies have well established the proapoptotic potential of miR-145 which can be exerted by TP53 activation. In turn, activation of TP53 will promote the expression of miR-145, which forms a death-stimulating loop involving miR-145 and TP53 [58]. The loop acts through the communication between the response element of p53 and the miR-145 promoter region [59]. c-Myc, as an oncogene, and its target genes, including eukaryotic translation initiation factor 4E (eIF4E) and Cyclin-Dependent Kinase 4 (CDK4), are silenced following the miR-145 upregulation, which leads to tumor suppression [60]. Besides, the upregulation of miR-145 is remarkably effective on ovarian cancer cells, inhibits their proliferation, migration, and invasion, and suppresses tumor growth and metastasis [61, 62]. Also, TP53 upregulates miR-145 expression and increases its effects on tumor suppression [58, 63, 64]. We showed a significant upregulation of TP53 in the miR, miR-MSC, and miR-MSC-Exo groups, whereas there was a significant superiority in increasing the TP53 mRNA level in the miR-MSC-Exo group. In line with our findings, a recent study on hepatocellular carcinoma (HCC) showed that in HCC cells with miR-145 overexpression, the expression level of p53 and p21 will enhance in comparison with control cells. p53 and p21 are considered the most critical checkpoints of the Gap1/Synthesis (G1/S) phase, which link with the complex of Cyclin-Dependent Kinase 6 (CDK6)/CyclinD1, interrupt with Cyclin-Dependent Kinases (CDKs), and consequently lead to cell cycle arrest [65]. Also, since the miR-145 binding sites are fit for the 3′-untranslated region (UTR) of the oncogene mouse double minute 2 (MDM2), it is regarded as a possible miR-145 target. In both HCC cells and tumor tissues, the mRNA level of MDM2 is significantly increased which is inversely associated with miR-145 expression [65]. Also, it is reported that miR-145 overexpression would decrease the expression level of MDM2 mRNA and protein. In controlling p53 protein stability, MDM2, as a ubiquitin ligase for p53, plays a central role [66].

According to previous studies, ROCK1 plays an important role in the regulation of actin cytoskeleton reorganization as a pivotal event during cell motion and invasion, and also its gene has recently been identified as a novel direct target of miR-145 [67, 68]. In the present study, a significant downregulation was observed in both the miR and miR-MSC-Exo groups which is supported by an in vitro study in which miR-145 overexpression in MCF-7 and BT-549 cell lines leads to downregulation of ROCK1 and subsequently inhibits cell proliferation, migration, and invasion [69]. The priority of the miR-MSC-Exo group in lowering the ROCK1 expression level can be attributed to the significantly higher efficiency of exosomes in transfecting miR-145 into T-47D cells than direct transfection.

Clearly, all the above gene expression patterns showed more efficiency for the miR-MSC-Exo group, which can be exerted for the better exosome-mediated delivery of miR-145. Similarly, Naseri et al. have reported the bone marrow-derived MSC-Exo as proper nanocarriers for therapies based on RNA applications [70]. Different studies using confocal laser scanning microscopy and quantitative reverse transcription-polymerase chain reaction (qRT-PCR) revealed the fact of joining and absorption of exosomes derived from MSCs with the lipid bilayer membrane of the breast cancer cells [70, 71]. Furthermore, an in vitro study on exosomes derived from MSCs including miR-100, as a tumor-suppressive miRNA, on MDA-MB-231 and MCF-7 cells indicated a decrease in the expression level of mTOR and HIF-1*α* and suppressed VEGF expression involved in angiogenesis control [72]. Moreover, the evaluation of the miR-124 overexpression in MSC exosomes has shown the proliferation inhibition of ovarian cancer cells by arrest induction in the S phase through the downregulation of Cyclin-Dependent Kinase 2 (CDK2), CDK4, and CDK6 [73].

## 5. Conclusion

In this study on the effect of miR-145 delivery on the breast cancer T-47D cell line, focusing on its roles in apoptosis induction and metastasis inhibition, we found positive results in both the direct and indirect miR-145-treated groups. However, exosome-mediated delivery of miR-145 caused an improved anticancer property of miR-145. Therefore, restoration of miR-145 by use of exosomes derived from mesenchymal stem cells can be considered a potential novel therapeutic strategy in breast cancer in the future.

## Figures and Tables

**Table 1:** Specific primers for target genes.

Name	Primer sequence (5′-3′)	Product size	Annealing melting template (°C)
ROCK1	F: TTTGAACAGGAAGGCGGAC	165	56
	R: GGGCGAAGAGGAAGACGA		

ERBB2	F: CTCGTCCCCCTGCTGTGT	178	59
	R: TGAACAGGACAGCAAAGGTTCT		

MMP9	F: GGTGATTGACGACGCCTTTG	133	60
	R: ATACCCGTCTCCGTGCTCC		

TP53	F: ATGATTTGATGCTGTCCCCG	221	58
	R: CAAGAAGCCCAGACGGAAAC		

miR-145	F: GGTCACTACTCCCCCCCAG	156	57
	R: GACAAGGTGGGAGCGAGTG		

## Data Availability

The data used to support the findings of this study are available from the corresponding author upon request.
